# Analytical characterization of optical solitons and bifurcation analysis for the (2+1)-D Wazwaz-Kaur Boussinesq equation

**DOI:** 10.1038/s41598-025-23216-3

**Published:** 2025-11-24

**Authors:** Islam Samir, Niveen Badra, Nesreen Sirelkhtam Elmki Abdalla, M. Elsaid Ramadan, Hamdy M. Ahmed

**Affiliations:** 1 Department of Mathematics and Engineering Physics, Faculty of Engineering, Aim Shams University, Cairo, Egypt; 2 Department of Mathematics, College of Science, King Khalid University, Abha, Saudi Arabia; 3 Department of Mathematics, Faculty of Science, Islamic University of Madinah, Medina, Saudi Arabia; 4 Department of Physics and Engineering Mathematics, Higher Institute of Engineering, El Shorouk Academy, Cairo, Egypt

**Keywords:** Solitary wave dynamics, Bright and dark solitons, Nonlinear evolution equations, Optical fiber systems, Bifurcation theory, Stability analysis, Mathematics and computing, Optics and photonics, Physics

## Abstract

In this paper, we address the analytical study of optical soliton solutions and the dynamical behavior of the (2+1)-dimensional Wazwaz-Kaur Boussinesq (WKB) equation, which models nonlinear wave propagation in higher-dimensional physical systems. Understanding such nonlinear structures is essential due to their relevance in modern optical fiber communications and nonlinear dispersive media. To tackle the problem, we employ the improved modified extended tanh function (IMETF) method, a symbolic analytical approach that efficiently generates a wide spectrum of exact solutions. Using this method, we successfully construct bright, dark, and singular soliton solutions, in addition to exponential and periodic wave structures. The analytical findings are further supported by comprehensive 2D, 3D, and contour graphical representations, which confirm the physical relevance and stability of the obtained solutions. Furthermore, we conduct a detailed bifurcation analysis to investigate the qualitative behavior of equilibrium points in the system. Phase portraits under various parameter settings illustrate how key parameters influence the creation, annihilation, and stability of these points. The novelty of this work lies in the combination of soliton solution construction with an in-depth bifurcation analysis for the WKB equation, which has not been previously explored in the literature. Our results extend existing studies by uncovering new types of solutions and providing insights into the system’s nonlinear dynamics, thereby contributing to the broader understanding of complex wave behavior in higher-dimensional nonlinear models.

## Introduction

In the context of nonlinear wave dynamics, the propagation of solitons in optical fibers is a fundamental phenomenon, with their stability and persistence proving invaluable for practical uses, notably in long-distance communication systems^1–8^. The pursuit of exact solutions to nonlinear partial differential equations is currently of significant interest due to their capacity to elucidate nonlinear phenomena. Consequently, numerous academics have devised diverse ways in recent years to derive exact solutions to nonlinear partial differential equations (NLPDEs). These approaches comprise the Riccati modified extended simple equation method^9^, the improved Bernoulli sub-equation function method^10^, modified Kudryashov’s method^11^, the Lie symmetry^12–14^, extended sinh-Gordon equation expansion approach^15,16^, modified F-expansion method^17^, modified simple equation method^18^ and the modified $\Big (\dfrac{G'}{G^2}\Big )$-expansion^19^.

The different kinds of soliton solutions must be taken into account in order to fully comprehend light propagation in optical fibers. Specifically, bright solitons, dark solitons, and periodic solitons play crucial roles in fiber optics, each with unique properties and applications^20–23^. Bright solitons are localized pulses of light that maintain their shape and intensity over long distances due to a delicate balance between dispersion and nonlinearity. These solutions are characterized by a peak in the light intensity, hence the name “bright” solitons. They are primarily used in applications that require high-intensity light beams. In contrast to bright solitons, dark solitons are characterized by a dip or notch in the light intensity. They arise in systems where the background light intensity is higher than the soliton itself. Dark solitons are advantageous in scenarios where background noise and intensity fluctuations need to be minimized. Periodic solitons, also known as breathers, exhibit oscillatory behavior in their intensity and shape. These solutions are intermediate between bright and dark solitons and can transform between the two states under certain conditions.

The (2 + 1)-dimensional WKB equation is a sophisticated mathematical model that extends traditional soliton theory into higher dimensions, capturing more complex wave behaviors that arise in optical fibers^24^. Despite the complexity of this equation, finding exact solutions is crucial for advancing our understanding of wave propagation in these systems. Studying this model have significant implications for both theoretical and applied physics. From a theoretical standpoint, the exact solutions enhance our comprehension of the underlying mathematical structures governing wave phenomena in higher dimensions^25^. Practically, these solutions provide significant potential for improving the design and optimization of optical communication systems, with soliton control and utilization contributing to better signal transmission and minimized loss over extended distances^26^.

In the current study, A. M. Wazwaz et al. presented novel variants of the Boussinesq equation across many spatial dimensions. A specific form of the model exists in (2 + 1)-dimensional space, commonly referred to as the (2 + 1)-dimensional WKB equation. The aforementioned equation is presented in^27^:

1 $$\begin{aligned} \mathcal {U}_{\text {tt}}-\mathcal {U}_{\text {xx}}-\varrho _1 \mathcal {U}^2{}_{\text {xx}}-\varrho _2 \mathcal {U}_{\text {xxxx}}+\frac{1}{4} \varrho _3^2 \mathcal {U}_{\text {yy}}+\varrho _3 \mathcal {U}_{\text {ty}}=0. \end{aligned}$$

The classical Boussinesq equation, which was first developed to describe the dynamics of long waves moving across shallow water surfaces, is modified by this equation. The classical Boussinesq equation accounts for both non linearity and dispersion, which are essential characteristics of wave behavior. There are numerous practical uses for the (2 + 1)-dimensional WKB equation, especially in domains involving fluid mechanics and wave dynamics. Six different families of solutions defined in terms of Jacobi elliptic functions were obtained by using the Jacobi elliptic function approach to this model in Ref^27^.. Bright solitons, singular solitons, and singular periodic solutions emerge as the Jacobi modulus is reduced to 0 or increased to 1. Ref^28^. utilized Hirota’s method along with the exponential expansion method to generate single soliton. This work aims to identify optical solitons and additional solutions utilizing the IMETF scheme. The mentioned method yields a novel and various types of solutions compared to alternative approaches. Among the derived solutions are singular, bright, and dark solitons, in addition to exponential and singular periodic forms.

This paper is structured as follows: Section 2 provides a synopsis of the suggested strategy. Section 3 demonstrates how the recommended method is applied to the model in order to obtain exact solutions. To visualize the behavior of the obtained solutions, Section 4 includes two-dimensional, three-dimensional, and contour graphical representations. Section 5 introduces bifurcation analysis to examine the stability of the system. Finally, the conclusion and the future work of are introduced in section 6.

## Summary approach

A concise elucidation of the IMETF scheme is obtained^29,30^. Considering the subsequent nonlinear partial differential equation:

2 $$\begin{aligned} \phi \left( \mathcal {U},~ \mathcal {U}_t,~ \mathcal {U}_{x},~\mathcal {U}_{y},~ \mathcal {U}_{xx},~ \mathcal {U}_{yy},,~\mathcal {U}_{xt}, ...\right) = 0, \end{aligned}$$

where $\phi$ is defined as a function of $\mathcal {U}$ and its corresponding partial derivatives.

In order to solve Eq.(2) utilizing the proposed methodology, the following procedures are executed:

*Step 1*: Assume

3 $$\begin{aligned} \mathcal {U}~(x,~y,~t)=\mathcal {H}(\xi ), \qquad \qquad \qquad \xi = x+ y-\eta ~ t, \end{aligned}$$

where $\eta$ is a constant such that $\eta \ne 0$. By substituting Eq. (3) into Eq. (2), the following ordinary differential equation is derived:

4 $$\begin{aligned} G(\mathcal {H},\mathcal {H}^\prime , \mathcal {H}^{\prime \prime }, \ldots ) = 0, \end{aligned}$$

*Step 2*: The solution corresponding to Eq. (4) is given below:

5 $$\begin{aligned} \mathcal {H}(\xi )=\sum _{i=-N}^N \alpha _i~ W(\xi )^i, \end{aligned}$$

the function $W(\xi )$ satisfies the auxiliary equation given below:

6 $$\begin{aligned} W'(\xi )=\sqrt{\tau _0+\tau _1 W(\xi )+\tau _2 W(\xi )^2+\tau _3 W(\xi )^3+\tau _4 W(\xi )^4}. \end{aligned}$$

*Step 3*: In the IMETF framework, the balancing principle is employed to determine the positive integer *N* in Eq. (5), which represents the highest power of $W(\xi )$ in the solution expansion. This is achieved by equating the order of the highest derivative term in Eq. (4) with the order of the highest nonlinear term, ensuring that the ansatz in Eq. (5) contains sufficient terms to capture all dominant contributions of the equation. This step is essential for the closure of the method, as it guarantees that the substituted series fully accommodates the nonlinear structure of the reduced ODE, leading to a solvable algebraic system for the coefficients $\alpha _i$ and parameters $\tau _j$.

*Step 4*: By inserting Eqs.(5) and (6) into Eq.(4), a polynomial expression in *W* is obtained. Equating the coefficients of corresponding powers to zero leads to a nonlinear system, which is subsequently solved using Mathematica.

*Step 5*: Eq. (6) admits the next general solutions:

**Case 1:** Setting $\tau _0 = \tau _1 = \tau _3 = 0$ leads to the following solutions:

$$\begin{aligned} W(\xi )=\sqrt{-\frac{\tau _2}{\tau _4}} \text {sech}\left( \sqrt{\tau _2} \xi \right) ,\quad \tau _2>0, \tau _4<0.\\ W(\xi )=\sqrt{-\frac{\tau _2}{\tau _4}} \sec \left( \sqrt{-\tau _2} \xi \right) , \quad \tau _2<0, \tau _4>0. \\ W(\xi )=\sqrt{-\frac{\tau _2}{\tau _4}} \csc \left( \sqrt{-\tau _2} \xi \right) , \quad \tau _2<0, \tau _4>0. \end{aligned}$$

**Case 2:** Setting $\tau _1 = \tau _3 = 0$ and $\tau _0 = \frac{\tau _2^2}{4\tau _4}$ leads to the following solutions:

$$\begin{aligned} W(\xi )=\sqrt{-\frac{\tau _2}{2 \tau _4}} \tanh \left( \sqrt{-\frac{\tau _2}{2}} \xi \right) ,\quad \tau _2<0, \tau _4>0. \\ W(\xi )=\sqrt{\frac{\tau _2}{2 \tau _4}} \tan \left( \sqrt{\frac{\tau _2}{2}} \xi \right) ,\quad \tau _2>0, \tau _4>0. \end{aligned}$$

**Case 3:** Setting $\tau _3 = \tau _4 = 0$ leads to the following solutions:

$$\begin{aligned} W(\xi )=\sqrt{\frac{\tau _0}{\tau _2}} \sinh \left( \sqrt{\tau _2} \xi \right) ,\quad \tau _2>0,\tau _0>0,\tau _1=0, \\ W(\xi )=\sqrt{\frac{-\tau _0}{\tau _2}} \sin \left( \sqrt{-\tau _2} \xi \right) ,\quad \tau _2<0,\tau _0>0,\tau _1=0, \\ W(\xi )=\exp \left( \sqrt{\tau _2} \xi \right) -\frac{\tau _1}{2 \tau _2},\quad \tau _2>0,\tau _0=\frac{\tau _1^2}{4 \tau _2}. \end{aligned}$$

**Case 4:** Setting $\tau _0 = \tau _1 = 0$ leads to the following solutions:

$$\begin{aligned} W(\xi )=-\frac{\tau _2 \left( \tanh \left( \frac{1}{2} \sqrt{\tau _2} \xi \right) +1\right) }{\tau _3},\quad \tau _3^2=4\tau _2\tau _4,\tau _2>0. \end{aligned}$$

Inserting the evaluated constants and general forms from Eq. (6) into Eq. (5) results in the derivation of various exact wave solutions.

## Analytic solutions

This section introduces the implementing of the proposed method on Eq. (1). Applying the transformation defined by Eq. (3) to Eq. (1) leads to the following reformulated equation:

7 $$\begin{aligned} -\mathcal {H}^{(4)} \varrho _2+\mathcal {H}'' \left( \eta ^2-\eta \varrho _3+\frac{\varrho _3^2}{4}-1\right) -2 \varrho _1 \left( \mathcal {H} \mathcal {H}''+\left( \mathcal {H}'\right) ^2\right) =0. \end{aligned}$$

When Eq. (7) is double integrated with regard to $\xi$ while ignoring the integration constants, we get:

8 $$\begin{aligned} -\mathcal {H}'' \varrho _2+\mathcal {H} \left( \eta ^2-\eta \varrho _3+\frac{\varrho _3^2}{4}-1\right) -\varrho _1 \mathcal {H}^2 =0. \end{aligned}$$

To determine the appropriate value of $N$ in the solution ansatz, we apply the balancing procedure to Eq. (8) by comparing the highest-order linear derivative term with the leading-order nonlinear term.

In Eq. (8), the second derivative $\mathcal {H}''$ contributes terms with dominant behavior of order $W^{N+2}$, due to the chain rule applied to the function $\mathcal {H}(\xi ) = \sum _{i=-N}^{N} \alpha _i W(\xi )^i$. On the other hand, the nonlinear term $\mathcal {H}^2$ yields terms of leading order $W^{2N}$.

By equating the dominant powers of $W(\xi )$ in both terms, we get:

$$N + 2 = 2N \quad \Rightarrow \quad N = 2.$$

Therefore, the value $N = 2$ is selected to ensure that all leading-order interactions between the linear and nonlinear terms are captured. As a result, the corresponding solution structure for Eq. (8) becomes:

9 $$\begin{aligned} \mathcal {H}(\xi ) = \alpha _{-2} W(\xi )^{-2} + \alpha _{-1} W(\xi )^{-1} + \alpha _0 + \alpha _1 W(\xi ) + \alpha _2 W(\xi )^2, \end{aligned}$$

the constants $\alpha _0$, $\alpha _1$, $\alpha _2$, $\alpha _{-1}$, and $\alpha _{-2}$ are defined, where it is required that either $\alpha _2$ or $\alpha _{-2}$ is nonzero.

Upon substituting Eqs. (9) and (6) into Eq. (8) and equating the coefficients of terms having identical powers to zero, the solutions for Eq. (1) are obtained in the following cases:

**Case 1**. $$\tau _0=\tau _1=\tau _3=0$$:

**Result 1**

$$\begin{aligned} & \alpha _0=0,~\alpha _1=0,~\alpha _2=-\frac{3 \tau _4 \left( 4 \eta ^2-4 \eta \varrho _3+\varrho _3^2-4\right) }{8 \tau _2 \varrho _1},~\alpha _{-1}=0,~\alpha _{-2}=0,~\tau _2=\frac{4 \eta ^2-4 \eta \varrho _3+\varrho _3^2-4}{16 \varrho _2}. \end{aligned}$$

Then, we have

10 $$\begin{aligned} & \mathcal {U}(x,y,t)=\frac{3 \left( 4 \eta ^2-4 \eta \varrho _3+\varrho _3^2-4\right) \text {sech}^2\left( \frac{1}{4} \sqrt{\frac{4 \eta ^2-4 \eta \varrho _3+\varrho _3^2-4}{\varrho _2}} (-\eta t+x+y)\right) }{8 \varrho _1}, \end{aligned}$$

11 $$\begin{aligned} & \mathcal {U}(x,y,t)=\frac{3 \left( 4 \eta ^2-4 \eta \varrho _3+\varrho _3^2-4\right) \sec ^2\left( \frac{1}{4} \sqrt{\frac{-4 \eta ^2+4 \eta \varrho _3-\varrho _3^2+4}{\varrho _2}} (-\eta t+x+y)\right) }{8 \varrho _1}, \end{aligned}$$

12 $$\begin{aligned} & \mathcal {U}(x,y,t)=\frac{3 \left( 4 \eta ^2-4 \eta \varrho _3+\varrho _3^2-4\right) \csc ^2\left( \frac{1}{4} \sqrt{\frac{-4 \eta ^2+4 \eta \varrho _3-\varrho _3^2+4}{\varrho _2}} (-\eta t+x+y)\right) }{8 \varrho _1}, \end{aligned}$$

Eq. (10) is a bright soliton while Eq. (11) and Eq. (12) represent singular periodic solutions.

**Result 2**

$$\begin{aligned} & \alpha _0=\frac{4 \eta ^2-4 \eta \varrho _3+\varrho _3^2-4}{4 \varrho _1},~\alpha _1=0,~\alpha _2=\frac{3 \tau _4 \left( 4 \eta ^2-4 \eta \varrho _3+\varrho _3^2-4\right) }{8 \tau _2 \varrho _1},~\alpha _{-1}=0,~\alpha _{-2}=0,\\ & \tau _2=\frac{-4 \eta ^2+4 \eta \varrho _3-\varrho _3^2+4}{16 \varrho _2}. \end{aligned}$$

Then, we have

13 $$\begin{aligned} & \mathcal {U}(x,y,t)=-\frac{\left( 4 \eta ^2-4 \eta \varrho _3+\varrho _3^2-4\right) \left( 3 \text {sech}^2\left( \frac{1}{4} \sqrt{\frac{-4 \eta ^2+4 \eta \varrho _3-\varrho _3^2+4}{\varrho _2}} (-\eta t+x+y)\right) -2\right) }{8 \varrho _1}, \end{aligned}$$

14 $$\begin{aligned} & \mathcal {U}(x,y,t)=-\frac{\left( 4 \eta ^2-4 \eta \varrho _3+\varrho _3^2-4\right) \left( 3 \sec ^2\left( \frac{1}{4} \sqrt{\frac{4 \eta ^2-4 \eta \varrho _3+\varrho _3^2-4}{\varrho _2}} (-\eta t+x+y)\right) -2\right) }{8 \varrho _1}, \end{aligned}$$

15 $$\begin{aligned} & \mathcal {U}(x,y,t)=-\frac{\left( 4 \eta ^2-4 \eta \varrho _3+\varrho _3^2-4\right) \left( 3 \csc ^2\left( \frac{1}{4} \sqrt{\frac{4 \eta ^2-4 \eta \varrho _3+\varrho _3^2-4}{\varrho _2}} (-\eta t+x+y)\right) -2\right) }{8 \varrho _1}. \end{aligned}$$

Eq. (13) is a bright soliton while Eq. (14) and Eq. (15) represent singular periodic solutions.

**Case 2**. $$\tau _0=\dfrac{\tau _2^2}{4\tau _4},~\tau _1=\tau _3=0$$:

**Result 1**

$$\begin{aligned} & \alpha _0=\frac{-4 \eta ^2+4 \eta \varrho _3-\varrho _3^2+4}{8 \varrho _1},~\alpha _1=0,~\alpha _2=-\frac{3 \tau _4 \left( 4 \eta ^2-4 \eta \varrho _3+\varrho _3^2-4\right) }{4 \tau _2 \varrho _1},~\alpha _{-1}=0,~\alpha _{-2}=0,\\ & \tau _2=\frac{4 \eta ^2-4 \eta \varrho _3+\varrho _3^2-4}{8 \varrho _2}. \end{aligned}$$

Then, we have

16 $$\begin{aligned} & \mathcal {U}(x,y,t)=-\frac{\left( 4 \eta ^2-4 \eta \varrho _3+\varrho _3^2-4\right) \left( 3 \tanh ^2\left( \frac{1}{4} \sqrt{\frac{-4 \eta ^2+4 \eta \varrho _3-\varrho _3^2+4}{\varrho _2}} (-\eta t+x+y)\right) +1\right) }{8 \varrho _1}, \end{aligned}$$

17 $$\begin{aligned} & \quad \mathcal {U}(x,y,t)=\frac{\left( 4 \eta ^2-4 \eta \varrho _3+\varrho _3^2-4\right) \left( 3 \tan ^2\left( \frac{1}{4} \sqrt{\frac{4 \eta ^2-4 \eta \varrho _3+\varrho _3^2-4}{\varrho _2}} (-\eta t+x+y)\right) -1\right) }{8 \varrho _1}. \end{aligned}$$

Eq. (16) gives a dark soliton solution, in contrast to the singular periodic behavior described by Eq. (17).

**Result 2**

$$\begin{aligned} & \alpha _0=\frac{-4 \eta ^2+4 \eta \varrho _3-\varrho _3^2+4}{8 \varrho _1},~\alpha _1=0,~\alpha _2=0,~\alpha _{-1}=0,~\alpha _{-2}=-\frac{3 \tau _2 \left( 4 \eta ^2-4 \eta \varrho _3+\varrho _3^2-4\right) }{16 \tau _4 \varrho _1},\nonumber \\ & \quad \tau _2=\frac{4 \eta ^2-4 \eta \varrho _3+\varrho _3^2-4}{8 \varrho _2}. \end{aligned}$$

Then, we have

18 $$\begin{aligned} & \mathcal {U}(x,y,t)=\frac{\left( 4 \eta ^2-4 \eta \varrho _3+\varrho _3^2-4\right) \left( 3 \coth ^2\left( \frac{1}{4} \sqrt{\frac{-4 \eta ^2+4 \eta \varrho _3-\varrho _3^2+4}{\varrho _2}} (-\eta t+x+y)\right) -1\right) }{8 \varrho _1},\end{aligned}$$

19 $$\begin{aligned} & \quad \mathcal {U}(x,y,t)=-\frac{\left( 4 \eta ^2-4 \eta \varrho _3+\varrho _3^2-4\right) \left( 3 \cot ^2\left( \frac{1}{4} \sqrt{\frac{4 \eta ^2-4 \eta \varrho _3+\varrho _3^2-4}{\varrho _2}} (-\eta t+x+y)\right) +1\right) }{8 \varrho _1}. \end{aligned}$$

Eq. (18) gives a singular soliton solution, in contrast to the singular periodic behavior described by Eq. (19).

**Result 3**

$$\begin{aligned} & \alpha _0=\frac{3 \left( 4 \eta ^2-4 \eta \varrho _3+\varrho _3^2-4\right) }{16 \varrho _1},~\alpha _1=0,~\alpha _2=\frac{3 \tau _4 \left( 4 \eta ^2-4 \eta \varrho _3+\varrho _3^2-4\right) }{16 \tau _2 \varrho _1},~\alpha _{-1}=0,\\ & \alpha _{-2}=\frac{3 \tau _2 \left( 4 \eta ^2-4 \eta \varrho _3+\varrho _3^2-4\right) }{64 \tau _4 \varrho _1},~\tau _2=\frac{-4 \eta ^2+4 \eta \varrho _3-\varrho _3^2+4}{32 \varrho _2}. \end{aligned}$$

Then, we have

20 $$\begin{aligned} & \mathcal {U}(x,y,t)=-\frac{3 \left( 4 \eta ^2-4 \eta \varrho _3+\varrho _3^2-4\right) \text {csch}^2\left( \frac{1}{4} \sqrt{\frac{4 \eta ^2-4 \eta \varrho _3+\varrho _3^2-4}{\varrho _2}} (-\eta t+x+y)\right) }{8 \varrho _1}, \end{aligned}$$

21 $$\begin{aligned} & \mathcal {U}(x,y,t)=\frac{3 \left( 4 \eta ^2-4 \eta \varrho _3+\varrho _3^2-4\right) \csc ^2\left( \frac{1}{4} \sqrt{\frac{-4 \eta ^2+4 \eta \varrho _3-\varrho _3^2+4}{\varrho _2}} (-\eta t+x+y)\right) }{8 \varrho _1}. \end{aligned}$$

Eq. (20) gives a singular soliton solution, in contrast to the singular periodic behavior described by Eq. (21).

**Case 3**. $$\tau _3=\tau _4=0$$:

**Result 1**

$$\begin{aligned} & \alpha _0=\frac{4 \eta ^2-4 \eta \varrho _3+\varrho _3^2-4}{4 \varrho _1},~\alpha _1=0,~\alpha _2=0,~\alpha _{-1}=0,~\alpha _{-2}=\frac{3 \tau _0 \left( 4 \eta ^2-4 \eta \varrho _3+\varrho _3^2-4\right) }{8 \tau _2 \varrho _1},\nonumber \\ & \tau _2=\frac{-4 \eta ^2+4 \eta \varrho _3-\varrho _3^2+4}{16 \varrho _2},~\tau _1=0. \end{aligned}$$

Then, we have

22 $$\begin{aligned} & \mathcal {U}(x,y,t)=\frac{\left( 4 \eta ^2-4 \eta \varrho _3+\varrho _3^2-4\right) \left( 3 \text {csch}^2\left( \frac{1}{4} \sqrt{\frac{-4 \eta ^2+4 \eta \varrho _3-\varrho _3^2+4}{\varrho _2}} (-\eta t+x+y)\right) +2\right) }{8 \varrho _1}, \end{aligned}$$

23 $$\begin{aligned} & \mathcal {U}(x,y,t)=-\frac{\left( 4 \eta ^2-4 \eta \varrho _3+\varrho _3^2-4\right) \left( 3 \csc ^2\left( \frac{1}{4} \sqrt{\frac{4 \eta ^2-4 \eta \varrho _3+\varrho _3^2-4}{\varrho _2}} (-\eta t+x+y)\right) -2\right) }{8 \varrho _1}. \end{aligned}$$

Eq. (22) produces a singular soliton, in contrast to Eq. (23), which leads to a periodic solution exhibiting singularities.

**Result 2**

$$\begin{aligned} & \alpha _0=0,~\alpha _1=0,~\alpha _2=0,~\alpha _{-1}=-\frac{3 \tau _1 \left( 4 \eta ^2-4 \eta \varrho _3+\varrho _3^2-4\right) }{4 \tau _2 \varrho _1},~\alpha _{-2}=-\frac{3 \tau _1^2 \left( 4 \eta ^2-4 \eta \varrho _3+\varrho _3^2-4\right) }{8 \tau _2^2 \varrho _1},\\ & \tau _2=\frac{4 \eta ^2-4 \eta \varrho _3+\varrho _3^2-4}{4 \varrho _2},~\tau _0=\frac{\tau _1^2}{4 \tau _2}. \end{aligned}$$

Then, we obtain the following exponential solution

24 $$\begin{aligned} & \mathcal {U}(x,y,t)=-\frac{3 \tau _1 \varrho _2 \exp \left( \frac{1}{2} \sqrt{\frac{4 \eta ^2-4 \eta \varrho _3+\varrho _3^2-4}{\varrho _2}} (-\eta t+x+y)\right) }{\varrho _1 \left( \exp \left( \frac{1}{2} \sqrt{\frac{4 \eta ^2-4 \eta \varrho _3+\varrho _3^2-4}{\varrho _2}} (-\eta t+x+y)\right) -\frac{2 \tau _1 \varrho _2}{4 \eta ^2-4 \eta \varrho _3+\varrho _3^2-4}\right) {}^2}. \end{aligned}$$

**Result 3**

$$\begin{aligned} & \alpha _0=\frac{4 \eta ^2-4 \eta \varrho _3+\varrho _3^2-4}{4 \varrho _1},~\alpha _1=0,~\alpha _2=0,~\alpha _{-1}=\frac{3 \tau _1 \left( 4 \eta ^2-4 \eta \varrho _3+\varrho _3^2-4\right) }{4 \tau _2 \varrho _1},\\ & \alpha _{-2}=\frac{3 \tau _1^2 \left( 4 \eta ^2-4 \eta \varrho _3+\varrho _3^2-4\right) }{8 \tau _2^2 \varrho _1},~\tau _2=\frac{-4 \eta ^2+4 \eta \varrho _3-\varrho _3^2+4}{4 \varrho _2},~\tau _0=\frac{\tau _1^2}{4 \tau _2}. \end{aligned}$$

Then, we obtain the following exponential solution

25 $$\begin{aligned} & \mathcal {U}(x,y,t)=\frac{\left( 4 \eta ^2-4 \eta \varrho _3+\varrho _3^2-4\right) \left( 1-\frac{12 \tau _1 \varrho _2 \left( 4 \eta ^2-4 \eta \varrho _3+\varrho _3^2-4\right) \exp \left( \frac{1}{2} \sqrt{\frac{-4 \eta ^2+4 \eta \varrho _3-\varrho _3^2+4}{\varrho _2}} (-\eta t+x+y)\right) }{\left( \left( 4 \eta ^2-4 \eta \varrho _3+\varrho _3^2-4\right) \exp \left( \frac{1}{2} \sqrt{\frac{-4 \eta ^2+4 \eta \varrho _3-\varrho _3^2+4}{\varrho _2}} (-\eta t+x+y)\right) +2 \tau _1 \varrho _2\right) {}^2}\right) }{4 \varrho _1}. \end{aligned}$$

**Case 4**. $$\tau _0=\tau _1=0$$:

**Result 1**

$$\begin{aligned} & \alpha _0=\frac{4 \eta ^2-4 \eta \varrho _3+\varrho _3^2-4}{4 \varrho _1},~\alpha _1=\frac{3 \tau _3 \left( 4 \eta ^2-4 \eta \varrho _3+\varrho _3^2-4\right) }{4 \tau _2 \varrho _1},~\alpha _2=\frac{3 \tau _3^2 \left( 4 \eta ^2-4 \eta \varrho _3+\varrho _3^2-4\right) }{8 \tau _2^2 \varrho _1},\\ & \alpha _{-1}=0,~\alpha _{-2}=0,~\tau _2=\frac{-4 \eta ^2+4 \eta \varrho _3-\varrho _3^2+4}{4 \varrho _2},~\tau _3^2=4\tau _2\tau _4. \end{aligned}$$

Then, we obtain the following dark soliton solution

26 $$\begin{aligned} & \mathcal {U}(x,y,t)=\frac{4 \eta ^2-4 \eta \varrho _3+\varrho _3^2-4}{8 \varrho _1}\Bigg \{-\frac{6 \tau _3 \varrho _2 \sqrt{\frac{\left( 4 \eta ^2-4 \eta \varrho _3+\varrho _3^2-4\right) {}^2}{\tau _3^2 \varrho _2^2}} \left( \tanh \left( \frac{1}{4} \sqrt{\frac{-4 \eta ^2+4 \eta \varrho _3-\varrho _3^2+4}{\varrho _2}} (-\eta t+x+y)\right) +1\right) }{4 \eta ^2-4 \eta \varrho _3+\varrho _3^2-4}\nonumber \\ & +3 \left( \tanh \left( \frac{1}{4} \sqrt{\frac{-4 \eta ^2+4 \eta \varrho _3-\varrho _3^2+4}{\varrho _2}} (-\eta t+x+y)\right) +1\right) {}^2+2\Bigg \}. \end{aligned}$$

## Graphic illustration

**Fig. 1:**
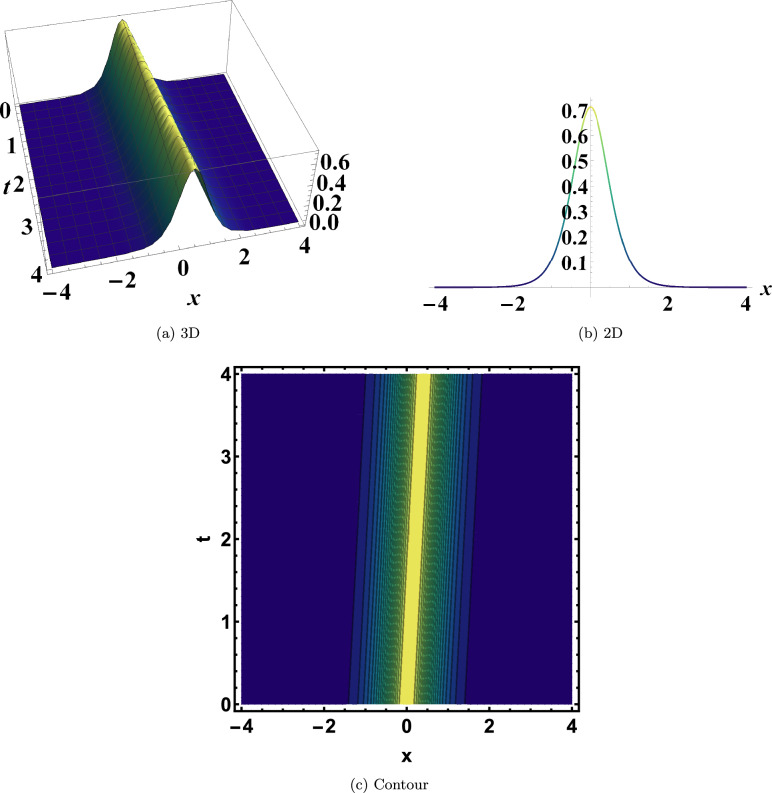
Bright soliton of Eq. (10). This bright soliton represents a localized wave packet of high intensity that maintains its shape and velocity, reflecting the stable energy transport enabled by the balance between nonlinearity and dispersion.

To demonstrate the physical characteristics of the extracted solutions, two-, three-, and contour-dimensional representations of several particular cases are provided. As shown in Fig. 1, Eq. (10) admits a bright soliton solution when the parameters are set to $y=0$, $\varrho _1=-2$, $\varrho _2=-0.095$, $\varrho _3=0.66$, and $\eta =0.105$. This bright soliton represents a localized wave packet of high intensity that maintains its shape and velocity, reflecting the stable energy transport enabled by the balance between nonlinearity and dispersion.

**Fig. 2:**
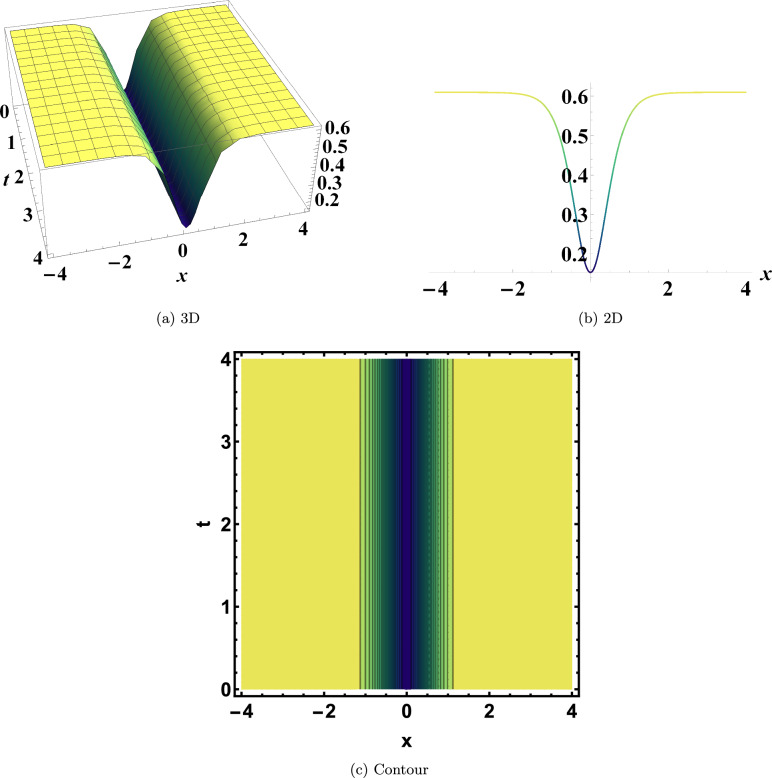
Dark soliton of Eq. (16). Physically, this dark soliton corresponds to a localized dip in intensity against a continuous wave background, a structure stabilized by the nonlinear–dispersive interplay.

As illustrated in Fig. 2, Eq. (16) yields a dark soliton solution when the parameters are set to 

$y=0$, $\varrho _1=2$, $\varrho _2=0.055$, $\varrho _3=1.33$, and $\eta =0.04$. Physically, this dark soliton corresponds to a localized dip in intensity against a continuous wave background, a structure that is also stabilized by the nonlinear–dispersive interplay.

**Fig. 3:**
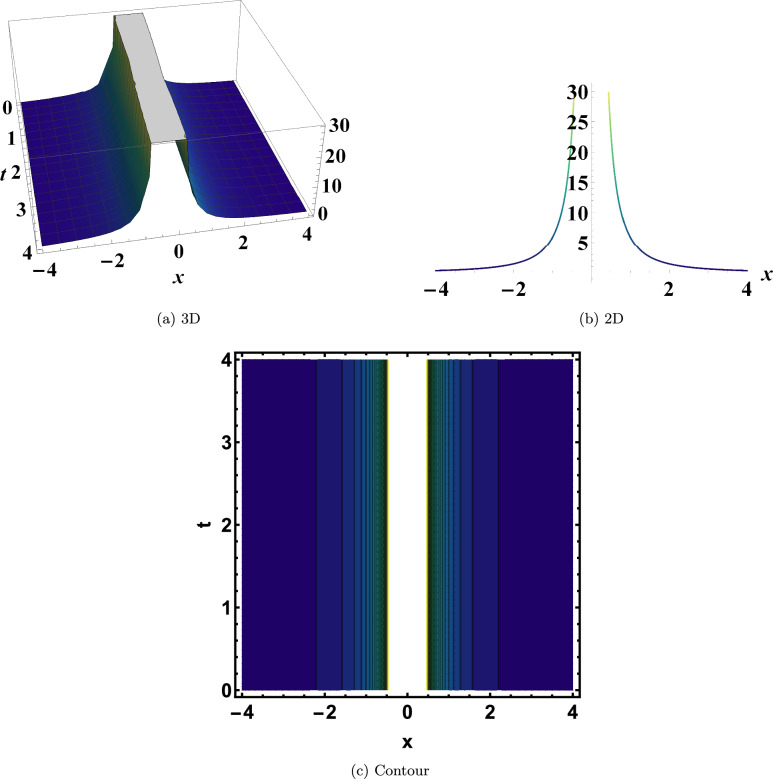
Singular soliton of Eq. (22). This rare soliton exhibits a sharp singularity or intensity spike, revealing the ability of nonlinear systems to generate exotic localized structures beyond standard solitary waves.

Figure 3 shows a singular soliton of Eq. (22) with $y=0,~\varrho _1=2,~\varrho _2=-2,~\varrho _3=-2,~\eta =-0.075$. This solution is remarkable because it describes a sharp singularity or spike in intensity, revealing how nonlinear systems can generate exotic localized structures beyond standard solitary waves.

**Fig. 4:**
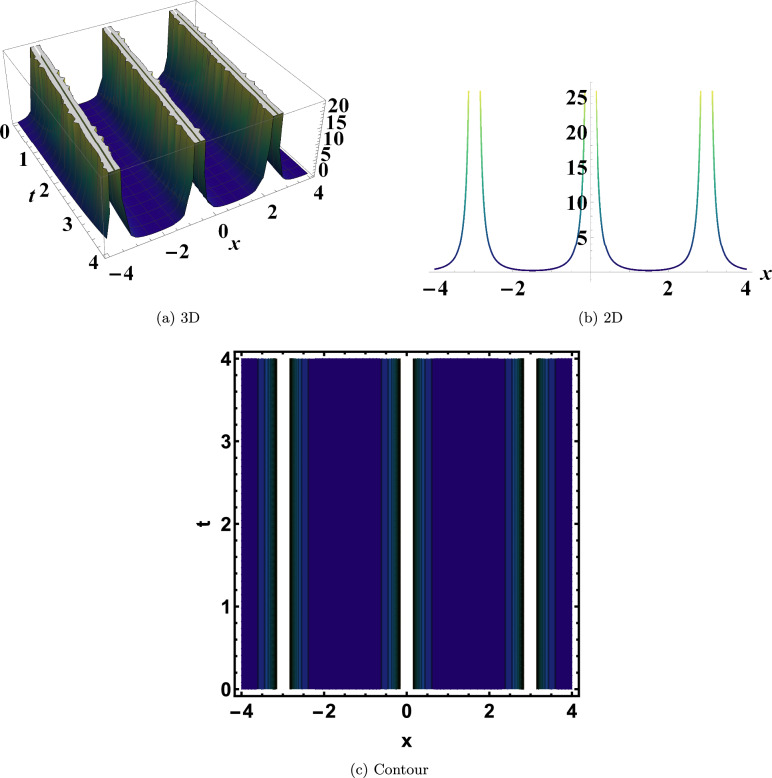
Singular periodic solution of Eq. (23). This solution combines periodic oscillations with singular features, providing insights into how nonlinear systems can sustain regularly recurring points of divergence.

As shown in Fig. 4, Eq. (23) produces a singular periodic solution when evaluated at $y=0$, $\varrho _1=2$, $\varrho _2=-0.195$, $\varrho _3=-1.065$, and $\eta =-0.16$. This structure combines periodic oscillations with singular features, offering important insights into how nonlinear systems can sustain regularly recurring points of divergence.

## Bifurcation analysis

Bifurcation analysis explores how the qualitative dynamics of a system evolve as certain parameters are varied. In nonlinear systems, even minor changes in parameters can trigger abrupt transitions in stability, equilibrium states, or periodic behaviors. These pivotal changes, known as bifurcations, often indicate the onset of phenomena such as oscillations, chaotic behavior, or multiple stable states^31,32^.

For the case of Eq. (8), the problem can be reformulated as follows:

27 $$\begin{aligned} \mathcal {H}''=L_1\mathcal {H}-L_2 \mathcal {H}^2, \end{aligned}$$

where

28 $$\begin{aligned} L_1=\dfrac{ \left( \eta ^2-\eta \varrho _3+\frac{\varrho _3^2}{4}-1\right) }{\varrho _2},~~~~~~ \text {and}~~~~~L_2=\dfrac{\varrho _1}{\varrho _2}. \end{aligned}$$

To transform the above equation into a planar first-order system, we introduce the substitution $\mathcal {H}'=q$, leading to the following system of first-order differential equations

29 $$\begin{aligned} \left\{ \begin{aligned}&\mathcal {H}'=q, \\&{q}'=L_1 \mathcal {H}-L_2 \mathcal {H}^2. \end{aligned} \right. \end{aligned}$$

By equating both $\mathcal {H}'$ and $q'$ to zero, we get the equilibrium points $(\mathcal {H},q)$. The equilibrium points are $\left( 0,0\right)$ and $\left( \dfrac{L_1}{L_2},0\right)$. To analyze the stability of the equilibrium points, we compute the Jacobian matrix associated with Eq. (29):

$$J = \begin{bmatrix} \frac{\partial f_1}{\partial \mathcal {H}} & \frac{\partial f_1}{\partial q} \\ \frac{\partial f_2}{\partial \mathcal {H}} & \frac{\partial f_2}{\partial q} \end{bmatrix} = \begin{bmatrix} 0 & 1 \\ L_1-2 L_2 \mathcal {H} & 0 \end{bmatrix} ,$$

where $f_1(\mathcal {H},q)=q$ while $f_2(\mathcal {H},q)=L_1 \mathcal {H}-L_2 \mathcal {H}^2$ and *J* has the following eigenvalues.

30 $$\begin{aligned} \lambda =\pm \sqrt{L_1-2 L_2 \mathcal {H}}. \end{aligned}$$

**Stability at** (0, 0)

In this case the eigen values will be $\lambda =\pm \sqrt{L_1}$. So when $L_1>0$, we get a real eigen values with opposite signs meaning that the equilibrium will be unstable (saddle point). When $L_1<0$, the eigenvalues will be purely imaginary meaning that the equilibrium point is a center (neutral stability, periodic orbits).

**Stability at** $$\left( \dfrac{L_1}{L_2},0\right)$$

In this case the eigen values will be $\lambda =\pm \sqrt{-L_1}$. So when $L_1<0$, we get a real eigen values with opposite signs meaning that the equilibrium will be unstable (saddle point). When $L_1>0$, the eigenvalues will be purely imaginary meaning that the equilibrium point is a center (neutral stability, periodic orbits).

we can see the stability at these equilibrium points in the trajectory in the following phase portraits.

**Fig. 5:**
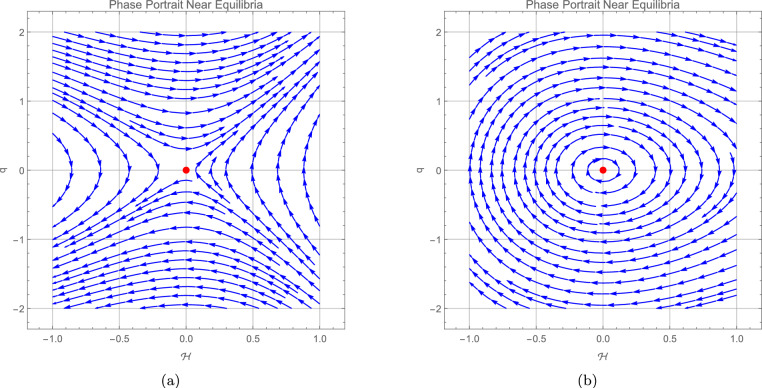
Represents the phase portrait around the (0, 0), where figure (**a**) admits an unstable saddle equilibrium point with $\eta =-0.732,\; \varrho _{1} = -1,\; \varrho _{2} = 1,\; \varrho _{3} = 2$, and figure (**b**) center neutrally stable equilibrium point with oscillatory behavior with $\eta =-0.732,~\varrho _1 = 1,~\varrho _2 = -1,~\varrho _3 = 2$.

**Fig. 6:**
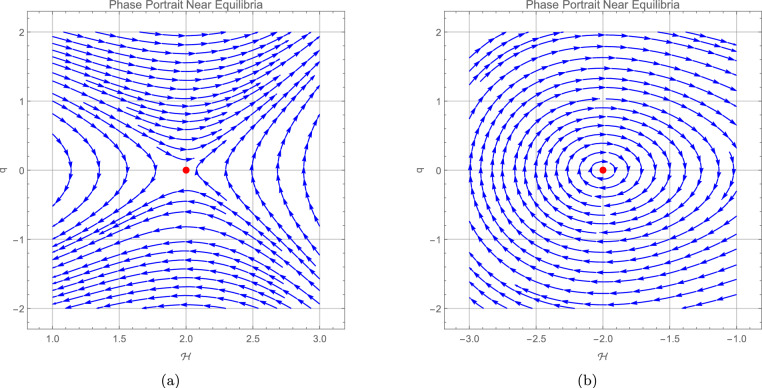
Represents the phase portrait around the $\left( \dfrac{L_1}{L_2},0\right)$, where figure (**a**) admits an unstable saddle equilibrium point with $\eta =-0.732,~\varrho _1 = 1,~\varrho _2 = -1,~\varrho _3 = 2$, and figure (**b**) center neutrally stable equilibrium point with oscillatory behavior for the parameter set $\eta =-0.732,\; \varrho _{1} = -1,\; \varrho _{2} = 1,\; \varrho _{3} = 2$.

It should be noted that the bifurcation analysis is not limited to phase portraits only. The parameters $L_1$ and $L_2$ defined in (28) are auxiliary quantities, while the actual bifurcation depends on the original system parameters $\eta ,~\varrho _3,~\varrho _1,$ and $\varrho _2$. Therefore, the figures presented here serve as illustrative phase portraits that reflect how the qualitative behavior of the system changes with variations in the original parameters. This clarifies that the bifurcation diagrams are directly linked to the physical parameters of the model, not only to the reduced forms $L_1$ and $L_2$. Figure 5 illustrates these results by showing the phase portraits around the equilibrium point (0, 0). In panel (a), with the parameter set $\eta =-0.732,\; \varrho _{1}=-1,\; \varrho _{2}=1,\; \varrho _{3}=2$, the trajectories form the typical structure of an unstable saddle equilibrium. In panel (b), for $\eta =-0.732,~\varrho _{1}=1,~\varrho _{2}=-1,~\varrho _{3}=2$, the trajectories are closed curves indicating a neutrally stable center with oscillatory behavior. These numerical phase portraits therefore provide visual confirmation of the theoretical stability analysis at (0, 0). Figure 6 presents the phase portraits around the equilibrium point $\left( \tfrac{L_1}{L_2},0\right)$. In panel (a), corresponding to the parameter set $\eta =-0.732,~\varrho _{1}=1,~\varrho _{2}=-1,~\varrho _{3}=2$, the trajectories diverge along the characteristic directions of a saddle, confirming the instability predicted when $L_1<0$. Panel (b), obtained for $\eta =-0.732,\; \varrho _{1}=-1,\; \varrho _{2}=1,\; \varrho _{3}=2$, exhibits closed orbits that reflect a neutrally stable center with oscillatory behavior, consistent with the theoretical result for $L_1>0$. These numerical simulations therefore reinforce the analytical stability analysis at $\left( \tfrac{L_1}{L_2},0\right)$.

## Conclusion

In this article, the nonlinear (2 + 1)-dimensional WKB equation (WKBE) was successfully investigated using the improved modified extended tanh function scheme, yielding a variety of solution types. These include dark solitons, bright solitons, singular solitons, singular periodic solutions and exponential solutions. The results are significant for understanding complex wave phenomena in various physical contexts. 2D and 3D graphical representations were depicted To better visualize the behavior and dynamics of these solutions. In comparison with the previously reported results in Refs^27,28^., which were limited to specific solution families such as bright solitons or elliptic structures, the present work offers a broader class of analytical solutions. By employing the IMETF method, we successfully derived novel forms including singular, bright, dark, and periodic solitons, many of which have not been reported before for this model. This confirms the versatility and strength of the proposed approach. Furthermore, unlike Refs^27,28^., the current work conducts a detailed bifurcation analysis, providing a qualitative understanding of the system’s equilibrium points and their stability under parameter variations. This examination elucidated the dependence of point emergence and disappearance on system parameters. Phase portraits were constructed for various parameter regimes, illustrating the system’s qualitative behavior. By manipulating and utilizing solitons, these technologies have the potential to improve optical communication system design and optimization, resulting in enhanced data transmission and decreased loss across long distances. This study not only contributes to the existing body of knowledge by providing new solutions to the WKBE but also sets the stage for further exploration of nonlinear equations in higher-dimensional systems. In the future, this approach may be expanded to investigate more generalized forms of the nonlinear (2 + 1)-dimensional WKBE, potentially uncovering additional solution structures or applications in engineering and physics. Future extensions could also consider the inclusion of perturbation terms or external forces to model more realistic scenarios in applied fields.

## Data Availability

The datasets used and/or analyzed during the current study are available from the corresponding author upon reasonable request.
